# MHCII3D—Robust Structure Based Prediction of MHC II Binding Peptides

**DOI:** 10.3390/ijms22010012

**Published:** 2020-12-22

**Authors:** Josef Laimer, Peter Lackner

**Affiliations:** Department of Biosciences, University of Salzburg, 5020 Salzburg, Austria

**Keywords:** bioinformatics, statistical scoring function, structure based binding prediction, MHC II peptide binding

## Abstract

Knowledge of MHC II binding peptides is highly desired in immunological research, particularly in the context of cancer, autoimmune diseases, or allergies. The most successful prediction methods are based on machine learning methods trained on sequences of experimentally characterized binding peptides. Here, we describe a complementary approach called MHCII3D, which is based on structural scaffolds of MHC II-peptide complexes and statistical scoring functions (SSFs). The MHC II alleles reported in the Immuno Polymorphism Database are processed in a dedicated 3D-modeling pipeline providing a set of scaffold complexes for each distinct allotype sequence. Antigen protein sequences are threaded through the scaffolds and evaluated by optimized SSFs. We compared the predictive power of MHCII3D with different sequence-based machine learning methods. The Pearson correlation to experimentally determine IC_50_ values for MHC II Automated Server Benchmarks data sets from IEDB (Immune Epitope Database) is 0.42, which is in the competitor methods range. We show that MHCII3D is quite robust in leaving one molecule out tests and is therefore not prone to overfitting. Finally, we provide evidence that MHCII3D can complement the current sequence-based methods and help to identify problematic entries in IEDB. Scaffolds and MHCII3D executables can be freely downloaded from our web pages.

## 1. Introduction

The binding of antigen peptides to class II MHC molecules is mandatory for their interaction with the T-cell receptor and the subsequent T-cell activation. The recognition of MHC II with a bound peptide by a T-cell drives canonical immune response. In misrouted immune responses, these interactions are associated with autoimmune diseases such as type 1 diabetes [1] or multiple sclerosis [2]. Moreover, MHC II presented peptides are considered as key in the immunotherapy to treat allergies [3]. Finally, for cancer immunotherapy, MHC II molecules and the MHC II pathway are of great importance [4]. Thus, identifying binding peptides—also referred to as the T-cell epitopes of an antigen—is currently an important target in basic research and clinical translation.

The mandatory step for any MHC II—T-cell interaction is the binding of a peptide. Different types of physical interactions between a MHC II molecule and a bound peptide at distinct positions along the binding pocket are responsible for specificity. Distinct pattern of polar, charged and hydrophobic residues are found in the different HLA gene loci of HLA-DP, HLA-DQ, HLA-DR (and the particular distinct HLA-DRB genes therein). Patronov et al. [5], for example, discussed this in detail for the HLA-DP2 allele. Numerous in vitro studies have been performed in order to determine properties of MHC II binders. Subsequently, computational models and methods have been developed to predict the binding of a certain peptide on a certain MHC II allotype.

The currently leading methods are based on the analysis of the sequence and the corresponding binding affinity data from numerous binding assay experiments. The first generation prediction methods SYFPEITHI [6] and RANKPEP [7] used sequence pattern and/or sequence profiles to represent binding (core) sequences in order to classify or rank antigen fragments. An extension to profiles are the so called quantitative matrices which include binding strength information [8,9]. Guan and coworkers [10] adopted a quantitative structure activity relationship (QSAR) concept for the prediction of IC_50_ values.

Later on, machine learning approaches such as neuronal networks [11], support vector machines [12] or decision trees [13], have been employed to infer the relationship between peptide sequence and binding strength with respect to a certain MHC II allotype. The idea of extrapolating binding specificity of experimentally uncharacterized MHC II allotypes from characterized ones by MHC II sequence similarity was finally implemented in the methods TEPITOPE [14], TEPTIOPEpan [15] and NetMHCIIpan [16].

With the increasing availability of structurally resolved MHC II molecules, a small number of structure-based methods have been developed. These methods are generally believed to be universal as they do not require specific experimental binding data for their setup. They can actually employ structure modeling approaches such as docking [17,18,19,20], threading [21,22] or molecular dynamics [22,23]. Zhang and coworkers assessed the principal limitations of structure-based methods [22] ending up with a moderate prognosis for the success of such methods compared to their sequence-based counterparts. However, the predictive power of the structure-based methods of Brodner [19] and Atanasova [20] is comparable to sequence-based approaches.

So far, structure-based methods did not proceed beyond the proof of principle. Here we present a new structure-based method, which allows for predicting of MHC II binding peptides for any allotype in runtime sufficient for large scale application with an accuracy comparable to the machine learning methods. For this purpose we employ template-based modeling to obtain a set of scaffold MHC II-peptide complex structures for each allotype. The scaffolds provide a simplified backbone representation of the MHC II-peptide complex, enabling an efficient threading procedure employing optimized statistical scoring functions (SSFs, a.k.a statistical energy function or knowledge-based potentials [24,25,26]). Here we follow the concept described in Sippl [24], which uses the Boltzmann distribution $P\left(r\right)=1/Ze^{-E(r)/kT}$ as basis and expresses the energy E(r) as a function of the probability distribution P(r)

(1) E(r)=−kTlnP(r)−kTlnZ.

Subsequently, the probability distribution P(r) is approximated by the observed distribution of distances *r*. The part −kTlnZ in the above equation is constant and can be omitted. The specific energy for a certain amino acid pair interaction then is

(2) $$\Delta E^{ab}\left(r\right)=-kT\ln f^{ab}\left(r\right)/f\left(r\right)$$

where $f^{ab}\left(r\right)$ is the distribution of the spatial distances *r* for a certain amino acid pair a,b and f(r) is the reference state, in our case the distribution of distances regardless of the involved amino acids pairs a,b. By summing up $\Delta E^{ab}\left(r\right)$ for a given sequence *S* and a given conformation *C*, the net energy ΔE(S,C) can be calculated. The prediction goal in this work is to determine which peptide sequences fit better into the binding groove than others. Thus we need to compare ΔE(S,C) values for different sequences *S*, which requires a further level of normalization. Sippl used an artificial poly-protein for this purpose [27]. As this is time-consuming and not easy to adapt for different purposes, we use here the statistic of the Wilcoxon-Mann-Whitney-Test as a discriminative score. Details are given in our previous work on protein stability prediction [28].

Using the Automated Server Benchmarks obtained from the Immune Epitope Database (IEDB) [29], we show that MHCII3D performs as well as the other methods reported therein. We demonstrate that MHCII3D is complementing other methods, such that the combined prediction performance of certain methods improves. We then compare MHCII3D with the leading method, NetMHCIIpan [30] on a benchmark data set provided by the authors of NetMHCIIpan 3.1 [31]. While the prediction accuracy of NetMHCIIpan is better on this test set, MHCII3D proves to be more robust in leave one molecule out tests.

## 2. Results and Discussion

In the following, first the prediction performance of our method in comparison and combination with other methods is shown, either on correlation or binary classification. We further briefly discuss the impact of binder/non-binder classifications regarding the chosen IC_50_ cutoffs and qualitative assessments provided by authors and IEDB submitters, respectively. The section closes with a discussion about the impact of peptide flacking regions on the prediction performance.

### 2.1. Prediction Performance

MHCII3D provides three quantities, a raw binding score, a rank or an IC_50_ value, which is denoted as MHCII3D-score, MHCII3D-rank and MHCII3D-IC50 in the figures and tables below. For binary classification, a cutoff value for predicted and experimentally determined IC_50_ needs to be applied in order to separate binders from non-binders. In the past, different authors defined different thresholds. For example, Wang et al. [32] apply a cutoff value of 1000 nM while Jensen et al. [31] use a value of 500 nM. For this work, we followed the definition of Jensen et al. and use a cutoff of IC_50_<500nM for binders in all classification experiments.

The performance of MHCII3D was tested on two data set: (i) the MHC II Weekly Benchmarks provided at the IEDB database (http://tools.iedb.org/auto_bench/mhcii/weekly/) and (ii) a set provided by Jensen et al. [31].

The IEDB database offers independent benchmark sets for MHC II binding data and prediction results for currently six methods, including a consensus method [32]. The methods are NetMHCIIpan-3.1 [16], NN-align [11], Comblib matrices [33], SMM-align [34], and Tepitope [14]. At irregular intervals, new benchmark sets are added based on newly added database entries. Table 1 summarizes the results of MHCII3D in comparison with the other method. Corresponding ROC plots are provided in supplementary file S1. Included are sets providing IC_50_ values and for which data are missing for at most one of the methods. The results for the full sets are provided in supplementary files S2 and S3. For all experiments on IEDB benchmarks, our IC_50_ estimation is based on a linear model derived from the data set provided by Jensen et al.

The results show that our method can provide predictions of similar quality to machine learning-based approaches. In two cases MHCII3D is better or as good as the best performing competitor.

**Figure 1:**
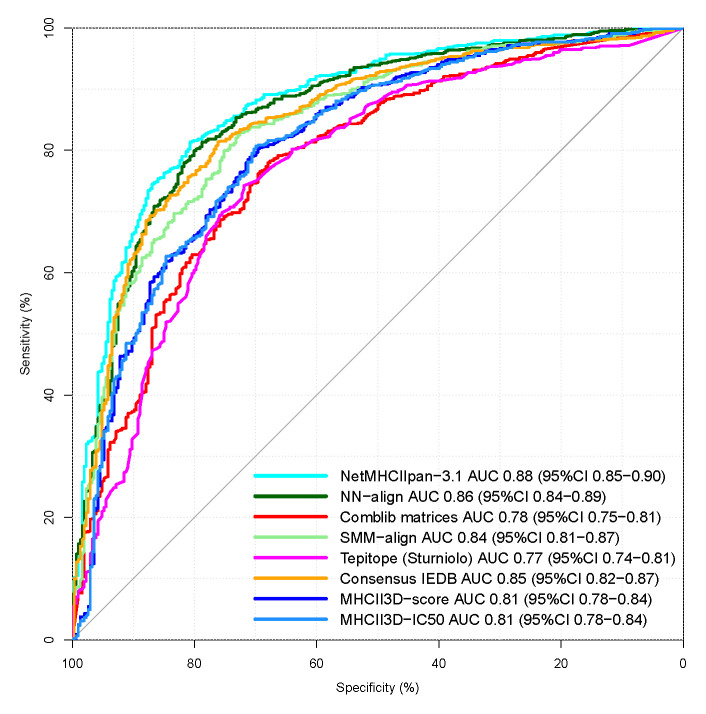
Classification performance of six existing prediction methods and our approach on a set of 1078 entries provided by Immune Epitope Database (IEDB) weekly benchmarks (2016-12-31–2019-03-22).

In addition to AUC values, IEDB also provides single prediction results for each entry and method. At the date of this analysis, nine of such benchmark sets are provided, containing predicted and measured IC_50_ values for 13,339 HLA-DR epitopes. Predictions from all six predictors are available for a subset of 1078 epitopes. Figure 1 shows the classification performance of our approach in comparison with the other methods listed in the data set. The MHCII3D based prediction achieves an AUC of 0.811 on this subset, which is in the range of the performance of the other six methods. Table 2 summarizes a comparison in terms of accuracy and false/true positive rates based on optimal thresholds, and Table 3 provides a statistical analysis of the ROC curves of Figure 1.

On this subset, MHCII3D achieves better results than the methods Comblib matrices and Tepitope but cannot reach the performance of the machine learning approaches (NetMHCIIpan.3.1, NN-align, and SMM-align).

The statistical analysis in Table 3 shows that our approach provides a significantly different prediction performance than most other methods, except for Comblib matrices.

We were also interested in the correlations between prediction results and experimentally determined IC_50_ values, respectively. Table 4 summarizes the correlations in terms of Pearson correlation coefficients (PCC, upper triangle) and Spearman’s rank correlation coefficients (SRCC, lower triangle).

Our approach achieves the second-highest PCC to the experimentally determined values; only NetMHCIIpan achieved a higher PCC. Further, MHCII3D predictions correlate quite well with the predictions of NetMHCIIpan and the IEDB Consensus.

The second evaluation set was utilized by Jensen et al. [31] in a five-fold cross-validation setup to show the classification performance of their method. We also evaluated our approach based on this setup. In opposite to various machine-learning techniques, the underlying statistical scoring functions (SSFs) of our method are not explicitly trained on specific binding data but are derived from a set of multimeric 3D protein structures omitting MHC II molecules. Thus, raw binding scores and rank values are not prone to overfitting on training values and are not affected by fold definitions in cross-validation setups. Estimated IC_50_ values, in contrast, depend on training data. Consequently, this value can overfit to a specific set of data. An n-fold cross-validation experiment can be used to reveal a tendency for overfitting. Jensen et al. also defined a leave-one-molecule-out (LOMO) experiment, based on the cross-validation setup, to show the prediction ability on uncharacterized alleles. Here all binding values for a certain allele are removed from the training sets, and predictions are only performed on this specific allele. Table 5 summarizes the results of these experiments for DRB alleles.

While the prediction performance is below those of NetMHCIIpan, the results also show the robustness of our approach. As mentioned above, the score and rank values are independent of the fold definitions and are therefore not reported separately for the LOMO experiment. It was shown in several studies related to the prediction of protein properties [28,31,36], that machine learning methods are prone to a reduced performance in such tests, so does NetMHCIIpan in this case. In contrast, the MHCII3D IC_50_ estimation results are stable, and results differ only marginally between the 5-fold and the LOMO experiments.

### 2.2. Qualitative versus Quantitative Measurement

**Figure 2:**
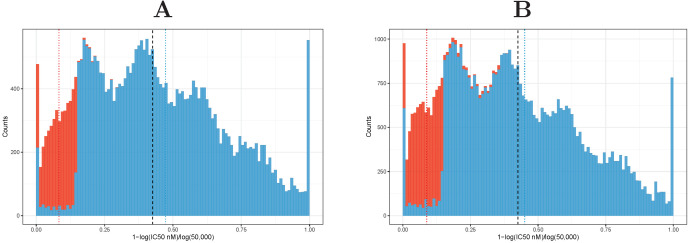
Qualitative vs. quantitative measurement. Distribution of log-transformed IC_50_ values grouped by qualitative measurement. Data are shown for (**A**) those entries of the data set provided by Jensen et al. [31], which can be mapped to the IEDB, and (**B**) the complete IEDB data set (HLA-DRB entries). IC_50_ values were transformed as described by Jensen et al. ($1-\log({\mathrm{IC}}_{50})/\log$(50,000)); therefore IC_50_ values >50,000 nM were set to 50,000 nM beforehand. Entries defined as binders (Positive, Positive-Low, Positive-Intermediate, Positive-High) are shown in blue, non-binder (Negative) are shown in red. Mean values are indicated as dotted lines; the black dashed lines indicate ${\mathrm{IC}}_{50}=500$.

During this work we noticed that repeatedly the qualitative assessment of an IEDB entry contradicts the presented quantitative measurement. For entries annotated as binders (positive), IC_50_ values as high as 500,000 nM are provided. On the other hand, negative entries with very low IC_50_ can be found. Figure 2 shows the distribution of IC_50_ for entries of the data set provided by Jensen et al. [31], which can be mapped to the IEDB and all currently provided HLA-DR entries in the IEDB data set.

We were able to map 35,529 entries between these two data sets. Based on this subset, we performed three additional five-fold cross-validation experiments to investigate the impact of different definitions for the classification. First, we evaluated the effect of the alternative binder/non-binder cutoff of ${\mathrm{IC}}_{50}=1000$ nM. In a second experiment, we validated our method based on the mapped qualitative IEDB assessment. In the final analysis, we only include those 21,724 entries where the qualitative assessment matches the classification based on an IC_50_ value of 500. Fold definitions were adopted from the experiments before. Table 6 summarizes the results of these experiments in comparison to a cross-validation based on the default IC_50_ threshold of 500 nM.

As shown in Table 6, the classification performance (AUC) increases with the percentage of peptides defined as binders. This effect can be partly explained by the higher portion of binders in the data set. Filtering for non-contradicting entries in the set leads to a clear separation of binder and non-binder as more than two-thirds of contradictions between IC_50_ value and qualitative measurements occur in a range between IC_50_ of 500 nM and 5000 nM (see supplementary file S4). This is also reflected in the achieved AUC values. Unfortunately, detailed per peptide/allele prediction data for other methods are not publicly available for this data set. Thus we were not able to analyze and compare these three experiments with competitor methods.

### 2.3. Consensus Prediction

As shown in Table 4, the predictions of our approach highly correlate with some existing methods and reach the second-highest PCC regarding the experimentally determined values. Consequently, it can be assumed that it complements those methods well. Wang et al. suggest a rank-based consensus prediction that utilizes the predictions of the three top-scoring methods. This allows to combine predictions of methods providing IC50 estimations and approaches with an alternative scoring on different scales. Thereby, the predictions of these methods are ranked for a specific set of peptides. Then the median rank for each peptide is computed, representing the consensus prediction [32].

At the time of this writing, the three top-scoring methods in the benchmark were NetMHCIIpan-3.1, NN-align, and SMM-align. Since we could not numerically reproduce the rank-based IEDB consensus prediction and the fact that IC_50_ values are provided for all of these methods, we adapted the consensus method by computing the median predicted IC_50_ value instead of a median rank. In the following, we show results based on this adaptation based on the three methods (Top3) and with our approach in replacement of SMM-align (Top2 + M), in comparison with the Consensus IEDB prediction (IEDB). This analysis is performed on the same subset of 1078 entries of the IEDB weekly benchmarks as described above. Table 7 summarizes the results for this experiment. The results for all IEDB benchmark sets providing prediction values for the top methods are presented in supplementary files S5 and S6.

The combination of our method with the two top-scoring tools shows the best performance in terms of classification (AUC) and regression (PCC, SRCC). Especially the PCC could be strongly improved (0.407 vs. 0.481) compared to the other consensus predictions.

**Figure 3:**
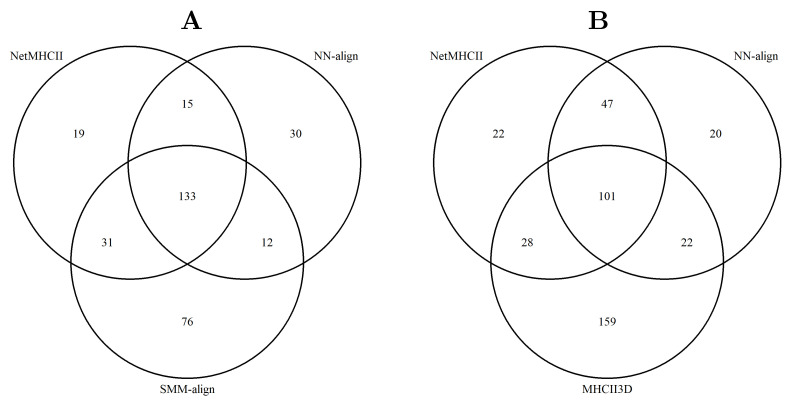
Outlier analysis based on a set of 1078 entries provided on the IEDB database. (**A**) Outliers among the top three performing prediction methods. (**B**) Outliers among the top two performing methods and MHCII3D.

A consensus method can improve the prediction performance by mitigating outliers of the underlying methods. Thereby, a key advantage of a median-based method is the higher robustness against those outliers. Consequently, such a consensus method only can improve the prediction performance if outliers are not common among methods. Figure 3 summarizes the results of an outlier analysis. For this, outliers were defined as follows: (i) The classification of an entry is wrong, meaning a peptide experimentally shown as a binder was predicted as a non-binder, and vice versa, (ii) and the prediction error (IC_50_) must be higher than 500 nM. By this, definition NetMHCIIpan-3.1 shows 198 outliers on the IEDB subset of 1078 entries, NN-align 190, SMM-align 252, and MHCII3D 310. Most of these outliers are common among the methods, where the outliers of our approach overlap slightly less with the machine learning methods than those of SMM-align.

### 2.4. Effect of Core Peptide Flanking Regions

In contrast to MHC I molecules, MHC II molecules have an open binding groove. Thus, peptides of variable lengths, mostly between 13 and 25 residues long [37], can bind to MHC II. Thereby, the binding affinity of a peptide to the MHC II complex is primarily determined by a nine-residues long binding-core but is also affected by the flanking residues [38,39].

**Figure 4:**
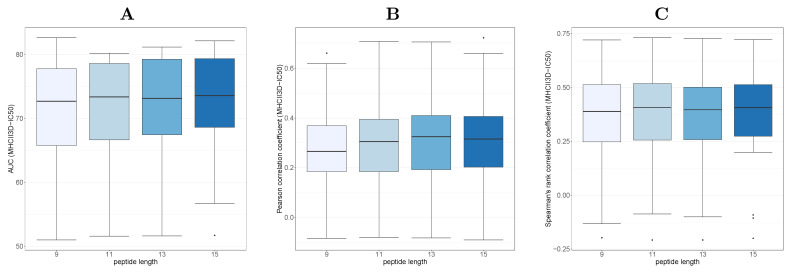
Prediction performance in terms of (**A**) classification (AUC), (**B**) Pearson’s correlation coefficient and (**C**) Spearman’s rank correlation coefficient for various binding peptide lengths in the MHC II models, grouped by MHC II alleles.

Consequently, we investigated the effects of various peptide lengths (9, 11, 13, and 15 residues long). Therefore, we placed peptide conformations of corresponding lengths in the binding groove of the MHC II models. The conformations were derived from known structures from the PDB, and superimposing was used to determine their positions within the binding grooves. Figure 4 summarizes the results of this analysis. The results show an improved prediction performance with longer peptide conformations. The Pearson’s correlation coefficient shows a maximum at peptide length 13. We finally use 15-mer peptides, as this had slightly better performance in the other tests.

### 2.5. Availability

MHCII3D is provided as a standalone version, available for Windows and Linux. can be freely downloaded from our web pages https://pbwww.che.sbg.ac.at/MHCII3D. Further, we provide a REST web service for the analysis of small data sets. An example script for Python for the access of the service is provided in Supplement S7.

## 3. Materials and Methods

### 3.1. Statistical Scoring Functions

Our approach is based on statistical scoring functions (SSFs) as implemented by MAESTRO [28]. Thereby, the prediction utilizes distance-dependent residue pair SSFs (pSSFs), scoring $C^{\alpha }$-$C^{\alpha }$ interactions and $C^{\beta }$-$C^{\beta }$ interactions, respectively. MAESTRO SSFs were initially designed to predict stability changes upon mutations, but have shown useful for other tasks. Here, interactions within the binding peptide and between the peptide and the MHC II complexes are scored. Special pSSFs were compiled to put a focus on interactions between distinct polypeptide chains. For this, we used a precompiled list from the PISCES database [40] (percentage identity: 50%, maximum resolution: 3.0, maximum R-value: 1.0). This set was then filtered for multimeric structures containing at least one polypeptide with a length between 5 and 20 residues, resulting in a list of 1227 PDB entries.

### 3.2. HLA-DR Models

3D models of MHC II complexes are required for our approach. In order to overcome the limitation to alleles with known, experimentally determined models, at least 100 models for each HLA-DR allele were generated utilizing the homology modeling tool MODELLER [41]. In the case of the availability of multiple, equally suitable template structures, models were generated based on each of them. All models include an alanine nonamer binding peptide as a placeholder, representing the peptide binding core.

Subsequently, the resulting models were scored with multiple scoring tools (DOPE [42], Rosetta [43], ProSa2003 [27], and MAESTRO [28]). The scores are then summarized to a model meta score. The top-scoring models were then utilized for predictions. All resulting models, including template structures, and scores are provided at our M23D database (https://pbwww.che.sbg.ac.at/m23d).

Template structures, required for homology modeling, were derived from PDB performing a BLAST search with the sequence of HLA-DRA*01:01. Subsequently, the resulting 168 PDB entries were checked by hand for structural errors, and any linkers between the MHC II complexes and binding peptides were removed according to the definitions provided by the corresponding publications. Finally, the chains in all template structures were renamed to the same scheme (chain A for the α-chain, chain B for the β-chain, and chain P for the binding peptide). During predictions, multiple peptide conformations are used. In order to enable an easy and fast substitution of placeholder with these alternative conformations, the binding pockets of the models are superimposed to reference PDB entry 4MDJ, which is the top-ranking template model in terms of resolution and R-factor.

### 3.3. Binding Score

The binding score is computed in four steps: (i) first, from the M23D database of HLA 3D models, the two models with the best meta score are selected for a certain HLA allele. Then, for each of these models, a set of five alternative peptide backbone conformations is derived from the modeling template structures. These alternative conformations replace the peptide placeholder in the models. Thus, small conformational varieties were obtained, which increase the prediction performance compared to a static model approach (see Supplementary File S8). This approach allows utilizing various sizes of peptide conformations without requiring new models of the main complex. (ii) The potential peptide sequence is then applied to each model and peptide conformation, respectively, and a pSSF score is computed. Thereby, the target sequence is slid through the peptide conformations, and scores are computed for each position covering at least the 9-mer core. (iii) Subsequently, the best fitting position of the sequence is determined based on these scores. (iv) Finally, a consensus is calculated by averaging the scores of each selected combination of model and peptide conformation.

### 3.4. Binding Rank

In addition to the raw binding score, an easy to interpret binding rank is implemented, similar to the rank value provided by NetMHCII. The rank ranges between 0.00 and 1.00, where low values indicate a binder. The binding rank compares a peptide score to background scores based on a set of 11,353 peptide sequences sampled from a non-redundant data set derived from PISCES database [40] (percentage identity: 20%, maximum resolution: 3.0, maximum R-value: 1.0).

### 3.5. IC_50_ Estimation

A linear model is used to convert the above described binding rank into an IC_50_ estimation. Thereby, a linear regression between log-transformed IC_50_ values listed in the data set provided by Jensen et al. [31] and the corresponding binding ranks (*r*) was used, resulting in the following equation for the MHCII3D-IC_50_ estimation:

(3) $$IC_{50pred.}=50000^{1-(-0.4265748\;r+0.51225)}$$

### 3.6. Validation Data Sets

Performance tests were performed on two data sets. For the first test set we use the weekly benchmark sets provided by the Immune Epitope Database (IEDB) [44]. At the time of this writing the sets contain binding values for in sum 13,927 epitopes and 21 alleles, respectively.

The second set published by Jensen et al. [31] includes 87,364 experimental determined IC_50_ binding values for 36 HLA-DR alleles. All IC_50_ values in this set are presented log-transformed, as described by Nielsen et al. [45]. The set was also used to compute a linear model for the estimation of IC_50_ values (see above). For binary classification testing, an IC_50_ threshold of 500 nM was used.

We also derived a subset of 35,529 entries from this data set, which can be mapped to the IEDB database, based on the given allele, peptide, and IC_50_ value. On this subset we performed various experiments regarding the binder/non-binder definition. We further removed cases where the quantitive value contradicts the qualitative label assigned by the authors. Thereby an IC_50_ threshold of 500 nM was used, and we did not distinguish between the different levels of positive labels (positive, positive-low, positive/intermediate, and positive-high). This "non-contradicting" set includes 21,724 entries.

### 3.7. Statistical Analysis

For statistical analyses, we utilized multiple software tools: R [46], and its package pROC [47], was used for the computations of AUC values and correlation coefficients. Plots were generated using the R package ggplot2 [48]. For a more detailed analysis, we used the StAR [35] web service (http://melolab.org/star/), which in addition to a general analysis of the prediction performance, provides an implementation of a nonparametric test by De Long et al. [49] for comparing ROC curves.

## 4. Conclusions

With MHCII3D we can show that the structure-based prediction of MHCII binding peptides is competitive to comparable sequence-based methods. On average, the predictive power of MHCII3D is lower than that of the leading machine learning methods, but still higher than that of other competitors. There is considerable room for improvement of our method regarding the prediction of binding affinities. On the other hand, MHCII3D is able to improve a consensus-based prediction method and thus complements the existing approaches.

In order to improve the prediction of binding affinities we aim to add more structural variability to the scaffolds by introducing local structural movements, smaller ones within and larger ones outside the 9-mere core region. We also plan to investigate if the utilization of (predicted) properties of the binding peptide itself is beneficial.

So far, MHCII3D and other methods concentrate on affinity prediction. Recently, several attempts have be made to include mass spectrometry data from MHC eluted ligands [30,50] in order to improve machine learning models for binary classification of binding peptides. Thereby, information about in vivo processed antigens is incorporated in the models which shifts the prediction from potential binders to biologically relevant binders.

Taking these different aspects into account, i.e., complementarity of MCHII3D, the performance of machine learning approaches and availability of novel experimental data, we will next focus on the integration of these components in order to further improve the predictive power of our method.

## Figures and Tables

**Table 1:** Classification performance (AUC) in comparison with other methods on the IEDB weekly benchmarks. Numbers are shown for IEDB references providing IC_50_ values and predictions are included for at least five of the pre-existing methods. ^a^ Result values are derived from the IEDB database (download version, http://tools.iedb.org/auto_bench/mhcii/weekly/).

Dataset & IEDB- Reference/ Allele	#Peptides	#Binder	NetMHCII- Pan-3.1 ^a^	NN-Align ^a^	SMM-Align ^a^	Comblib Matrices ^a^	Tepitope ^a^	Consensus IEDB ^a^	MHCII3D- IC50
**2016-12-31**—1028243									
DRB1*04:04	861	468	0.861	0.798	0.784	−	0.827	0.803	0.800
**2016-12-31—1028242**									
DRB1*03:01	863	492	0.855	0.777	0.776	−	0.747	0.788	0.727
**2016-12-31—1028241**									
DRB1*01:01	885	642	0.890	0.876	0.849	0.789	0.815	0.864	0.819
**2016-12-31—1028057**									
DRB1*01:01	29	22	0.890	0.851	0.753	0.851	0.740	0.851	0.773
DRB1*04:01	29	25	0.920	0.870	0.770	−	0.525	0.760	0.710
DRB1*07:01	29	27	0.889	0.907	0.796	0.778	0.722	0.963	0.722
DRB1*15:01	29	26	0.679	0.692	0.705	−	0.699	0.744	0.487
**2016-12-31—1027578**									
DRB1*03:01	14	10	1.000	0.975	0.900	−	0.925	0.950	0.775
DRB1*07:01	19	12	0.929	0.952	0.976	0.964	0.899	1.000	0.845
DRB3*01:01	20	7	0.945	0.901	0.879	0.703	−	0.846	0.571
DRB4*01:01	14	4	0.800	0.600	0.725	0.725	−	0.650	0.725
**2017-11-24—1032311**									
DRB1*01:01	16	14	1.000	0.964	1.000	0.589	1.000	0.964	1.000
**2018-11-23—1029531**									
DRB1*01:01	11	4	1.000	0.857	0.661	0.429	0.500	0.929	0.714
**2019-03-22—1034502**									
DRB1*03:01	21	3	0.907	0.759	0.870	−	0.611	0.796	0.463
DRB1*08:02	21	5	0.900	1.000	0.912	−	0.688	0.950	0.925
DRB1*11:01	21	5	0.912	0.825	0.863	−	0.787	0.863	0.700
DRB1*15:01	21	4	0.853	0.882	0.794	−	0.882	0.882	0.941

**Table 2:** Prediction accuracy (ACC) compared to other methods on a set of 1078 entries of the IEDB weekly benchmarks. Numbers are computed based on an optimal threshold (OT) utilizing the software tool StAR [35]. Besides the AUC and the accuracy, the false positive (fp), true positive (tp), the number of non-binders (N), and the number of binders (P) are shown. * In the case of Tepitope, the AUC value is inverted (1-AUC).

Classifier	AUC	ACC	OT	fp	tp	N	P
NetMHCIIpan.3.1	0.875	0.833	1490.73	0.314	0.891	306	772
NN-align	0.862	0.823	1200.40	0.330	0.883	306	772
SMM-align	0.841	0.803	1672.00	0.415	0.890	306	772
Comblib matrices	0.782	0.771	25,138.90	0.513	0.883	306	772
Tepitope *	0.773	0.777	−3.32	0.546	0.905	306	772
Consensus IEDB	0.849	0.810	69.11	0.431	0.905	306	772
MHCII3D-IC50	0.811	0.794	2248.70	0.484	0.904	306	772

**Table 3:** Statistical analysis of the ROC curves presented in Figure 1. The prediction methods are compared in terms of ΔAUC values (upper triangle) and *p*-values (lower triangle), computed by the StAR [35] software tool.

	NetMHCII- Pan-3.1	NN-Align	SMM-Align	Comblib Matrices	Tepitope	Consensus IEDB	MHCII3D- IC50
NetMHCII	−	0.013	0.034	0.092	0.102	0.027	0.064
NN-align	0.015	−	0.021	0.079	0.088	0.013	0.051
SMM-align	$3.358\times 10^{-8}$	0.003	−	0.059	0.067	0.008	0.030
Comblib	$4.326\times 10^{-16}$	$6.066\times 10^{-11}$	$9.136\times 10^{-7}$	−	0.009	0.066	0.029
Tepitope	$1.704\times 10^{-11}$	$4.226\times 10^{-8}$	$1.602\times 10^{-5}$	0.635	−	0.075	0.038
Cons. IEDB	$1.114\times 10^{-5}$	0.032	0.236	$5.020\times 10^{-11}$	$1.728\times 10^{-7}$	−	0.037
MHCII3D	$5.015\times 10^{-9}$	$1.849\times 10^{-5}$	0.017	0.051	0.024	0.002	−

**Table 4:** Correlations between prediction methods and experimentally determined IC_50_ values, respectively. Based on 1078 of 13,339 entries provided by IEDB weekly benchmarks (31 December 2016–22 March 2019). Upper triangle: Pearson correlation coefficient; lower triangle: Spearman’s rank correlation coefficient.

	Exp. IC_50_	NetMHCII- pan-3.1	NN-Align	SMM-Align	Comblib Matrices	Tepitope (Sturniolo)	Consensus IEDB	MHCII3D- IC50
Exp. IC_50_	−	0.486	0.395	0.306	0.278	−0.332	0.407	0.423
NetMHCII	0.755	−	0.858	0.773	0.600	−0.458	0.673	0.752
NN-align	0.723	0.931	−	0.807	0.583	−0.384	0.640	0.580
SMM-align	0.675	0.910	0.915	−	0.456	−0.382	0.510	0.461
Comblib	0.559	0.772	0.739	0.755	−	−0.281	0.551	0.399
Tepitope	−0.519	−0.616	−0.615	−0.637	−0.475	−	−0.619	−0.417
Cons. IEDB	0.687	0.900	0.928	0.924	0.808	−0.719	−	0.534
MHCII3D	0.568	0.734	0.690	0.668	0.637	−0.558	0.704	−

**Table 5:** Comparison of prediction performances between NetMHCIIpan-3.2 and MHCII3D. ^a^ Values derived from Jensen et al. [31]. Results for a five-fold cross-validation experiment (5-fold) and a leave-one-molecule-out (LOMO) experiment, as defined by Jensen et al. are shown.

			NetMHCIIpan-3.2		MHCII3D-	MHCII3D-	MHCII3D-IC50	
**Molecule**	**#Peptides**	**#Binders**	**5-Fold ^a^**	**LOMO ^a^**	**Score**	**Rank**	**5-Fold**	**LOMO**
DRB1*01:01	10,412	6376	0.832	0.783	0.693	0.695	0.693	0.693
DRB1*01:03	42	4	0.678	0.711	0.592	0.566	0.572	0.566
DRB1*03:01	5352	1457	0.816	0.699	0.596	0.596	0.594	0.594
DRB1*04:01	6317	3022	0.809	0.766	0.602	0.603	0.597	0.597
DRB1*04:02	53	19	0.701	0.789	0.625	0.630	0.625	0.625
DRB1*04:03	59	14	0.841	0.862	0.630	0.635	0.629	0.629
DRB1*04:04	3657	1852	0.812	0.791	0.682	0.684	0.679	0.679
DRB1*04:05	3962	1654	0.827	0.799	0.677	0.679	0.672	0.672
DRB1*07:01	6325	3456	0.875	0.830	0.712	0.716	0.710	0.710
DRB1*08:01	937	390	0.844	0.804	0.714	0.715	0.718	0.718
DRB1*08:02	4465	2036	0.834	0.765	0.646	0.650	0.635	0.637
DRB1*09:01	4318	2164	0.833	0.791	0.699	0.700	0.697	0.697
DRB1*10:01	2066	1521	0.923	0.905	0.744	0.745	0.735	0.736
DRB1*11:01	6045	2667	0.864	0.767	0.692	0.693	0.691	0.691
DRB1*12:01	2384	759	0.868	0.800	0.728	0.729	0.730	0.730
DRB1*13:01	1034	520	0.857	0.731	0.720	0.720	0.722	0.722
DRB1*13:02	4477	2249	0.885	0.701	0.647	0.649	0.648	0.648
DRB1*15:01	4850	2107	0.834	0.780	0.725	0.726	0.725	0.725
DRB1*16:02	1699	989	0.883	0.866	0.696	0.697	0.688	0.688
DRB3*01:01	4633	1415	0.888	0.801	0.614	0.615	0.609	0.609
DRB3*02:02	3334	1055	0.869	0.756	0.648	0.648	0.640	0.641
DRB3*03:01	884	510	0.840	0.734	0.737	0.737	0.738	0.738
DRB4*01:01	3961	1540	0.822	0.726	0.662	0.662	0.663	0.663
DRB4*01:03	846	525	0.841	0.794	0.733	0.734	0.736	0.736
DRB5*01:01	5125	2430	0.849	0.765	0.654	0.655	0.654	0.654
Average			0.837	0.781	0.675	0.675	0.672	0.672
Median			0.841	0.785	0.679	0.681	0.676	0.676

**Table 6:** Effect of various classification definitions. In literature, no consistent IC_50_ cutoff is used to define a peptide as a binder. Further, the qualitative label assigned by authors often contradicts the quantitative value. This table shows the prediction performance on 35,529 IEDB entries on various binder definitions: ^a^ IC_50_ cutoff 500 nM, ^b^ IC_50_ cutoff 1000 nM, ^c^ quality assessment from IEDB. ^d^ Performance on non-contradicting database entries (IC_50_ threshold = 500 nM). The number of available entries is reduced to 21,724 in this case.

		IC_50_ = 500 ^a^		IC_50_ = 1000 ^b^		IEDB Qual. ^c^		Non-Contradicting ^d^		
**Molecule**	**#Pep.**	**#Bind.**	**AUC**	**#Bind.**	**AUC**	**#Bind.**	**AUC**	**#Pep.**	**#Bind.**	**AUC**
DRB1*01:01	7493	4553	0.697	5201	0.699	6783	0.740	5270	4553	0.785
DRB1*01:03	40	3	0.586	4	0.542	40	−	3	3	−
DRB1*03:01	2246	589	0.622	786	0.629	1503	0.662	1323	583	0.699
**Molecule**	**#Pep.**	**#Bind.**	**AUC**	**#Bind.**	**AUC**	**#Bind.**	**AUC**	**#Pep.**	**#Bind.**	**AUC**
DRB1*04:01	2652	1186	0.616	1488	0.621	2346	0.690	1494	1184	0.728
DRB1*04:02	38	19	0.693	22	0.653	35	0.733	22	19	0.842
DRB1*04:03	59	14	0.635	23	0.591	53	0.726	20	14	0.810
DRB1*04:04	1185	584	0.695	703	0.723	1038	0.771	729	583	0.828
DRB1*04:05	1790	759	0.707	959	0.718	1542	0.787	1009	759	0.847
DRB1*07:01	2298	1116	0.731	1334	0.732	1962	0.781	1454	1116	0.836
DRB1*08:01	35	4	0.726	4	0.726	27	0.630	12	4	0.750
DRB1*08:02	1849	691	0.674	865	0.682	1445	0.690	1097	691	0.746
DRB1*09:01	1703	723	0.673	906	0.662	1468	0.659	961	723	0.723
DRB1*10:01	187	149	0.740	162	0.802	171	0.843	165	149	0.871
DRB1*11:01	2157	919	0.718	1116	0.728	1773	0.776	1306	919	0.836
DRB1*12:01	897	166	0.774	265	0.782	589	0.776	476	166	0.874
DRB1*13:01	144	40	0.732	44	0.743	76	0.732	108	40	0.788
DRB1*13:02	1940	749	0.656	925	0.663	1528	0.677	1162	749	0.725
DRB1*15:01	2361	980	0.733	1233	0.747	1934	0.757	1405	978	0.829
DRB1*16:02	129	74	0.531	97	0.623	127	0.819	76	74	0.845
DRB3*01:01	1641	276	0.575	422	0.569	1090	0.624	827	276	0.646
DRB3*02:02	858	119	0.699	168	0.707	438	0.726	539	119	0.776
DRB3*03:01	15	0	−	0	−	12	0.472	3	0	−
DRB4*01:01	1826	670	0.712	885	0.715	1465	0.759	1031	669	0.825
DRB4*01:03	3	3	−	3	−	3	−	3	3	−
DRB5*01:01	1983	907	0.697	1102	0.704	1662	0.760	1229	907	0.818
Sum	35,529	15,293		18,717		29,110		21,724	15,281	
Average			0.679		0.685		0.721			0.792
Median			0.696		0.705		0.733			0.818

**Table 7:** Performance of consensus predictions. Shown are the results reported by the IEDB benchmark service for the IEDB consensus method (IEDB), a consensus-based on the predictions of NetMHCIIpan-3.1, NN-align, and SMM-align (Top3) and consensus-based on the predictions of NetMHCIIpan-3.1, NN-align, and MHCII3D (Top2 + M).

			Classification (AUC)			PCC			SRCC		
**Allele**	**Entries**	**Binder**	**IEDB**	**Top3**	**Top2 + M**	**IEDB**	**Top3**	**Top2 + M**	**IEDB**	**Top3**	**Top2 + M**
DRB1*01:01	941	682	0.842	0.863	0.865	0.388	0.447	0.478	0.698	0.734	0.746
DRB1*07:01	137	90	0.924	0.931	0.932	0.576	0.492	0.556	0.749	0.787	0.794
all	1078	772	0.849	0.872	0.874	0.407	0.432	0.481	0.687	0.741	0.753

## Supplementary Materials

The supplementary materials are available online at https://www.mdpi.com/1422-0067/22/1/12/s1.
