# Supplementary material for: MHCII3D—Robust Structure Based Prediction of MHC II Binding Peptides
Source: Int J Mol Sci. 2020 Dec 22;22(1):12. doi: 10.3390/ijms22010012 (PMC7792572; doi:10.3390/ijms22010012)

S1 - Table 1 - ROC plots

ROC curves presenting the classification performance of six existing prediction methods and our approach on the data sets presented in table 1 (IEDB weekly benchmarks; 2016-12-31 - 2019-03-22).

S1a - excluding Comblib matrices

The following ROC curves include the results of table 1, where all methods (except Comblib matrices) provides prediction results (2869 entries).

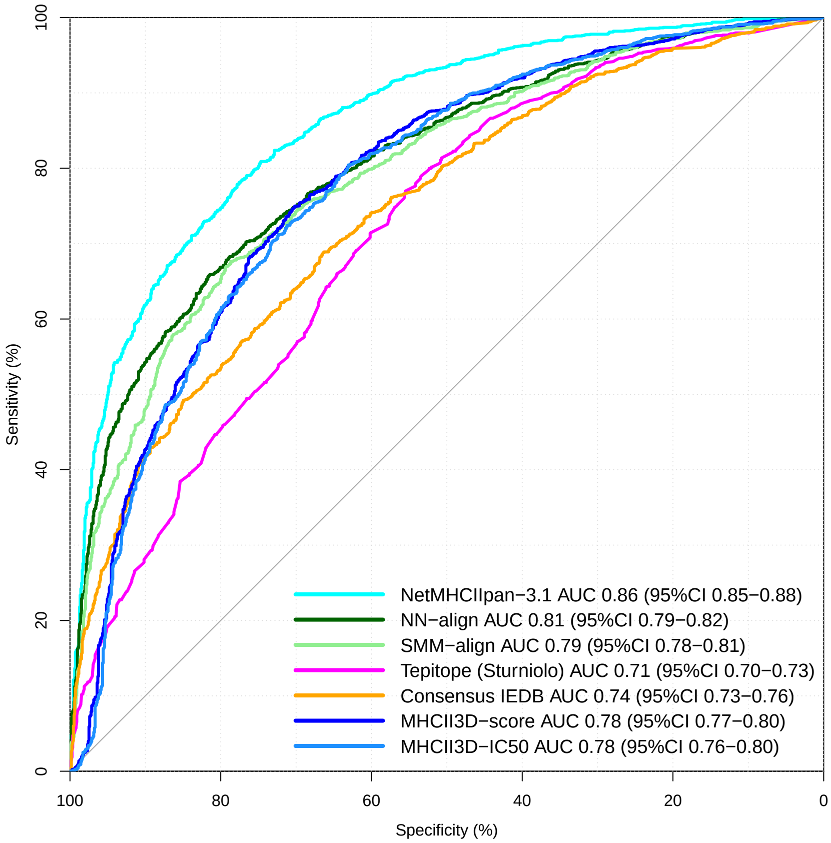

S1b - excluding Tepitope

The following ROC curves include the results of table 1, where all methods (except Tepitope) provides prediction results (1023 entries).

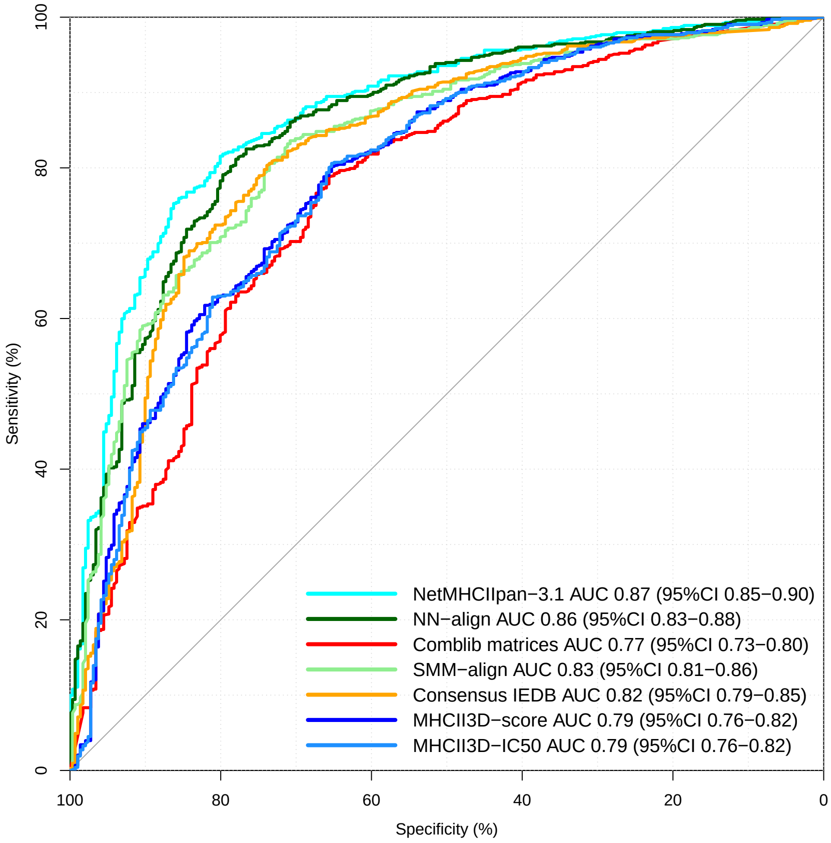

S1c - detailed ROC curves per weekly set/allele

2016-12-31 - 1028243

DRB1*04:04 - #peptides/#binder: 861/468

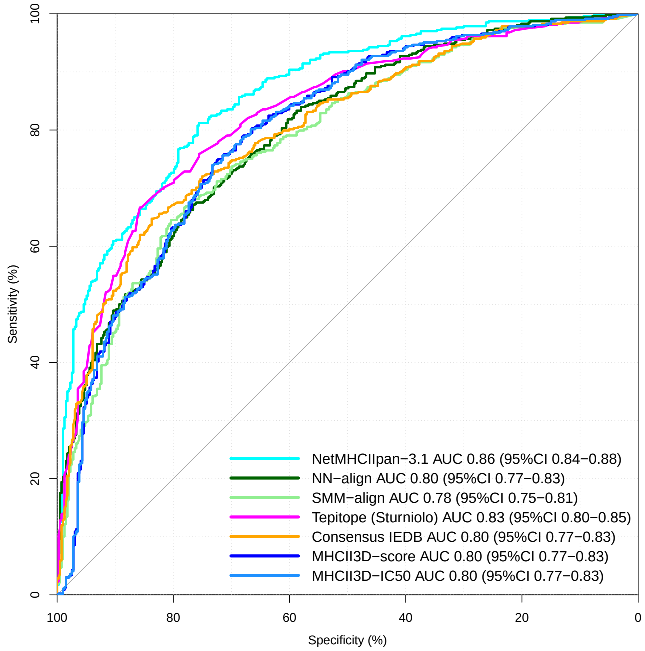

2016-12-31 - 1028242

DRB1*03:01 - #peptides/#binder: 863/492

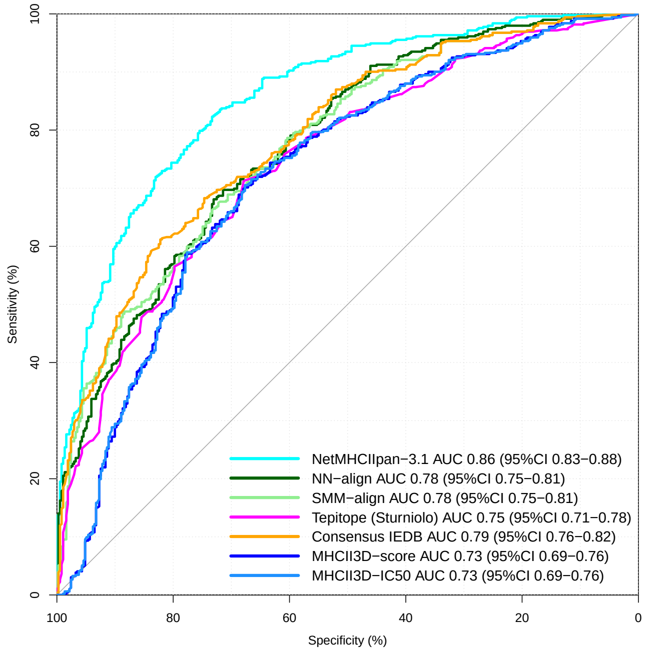

2016-12-31 - 1028241

DRB1*01:01 - #peptides/#binder: 885/642

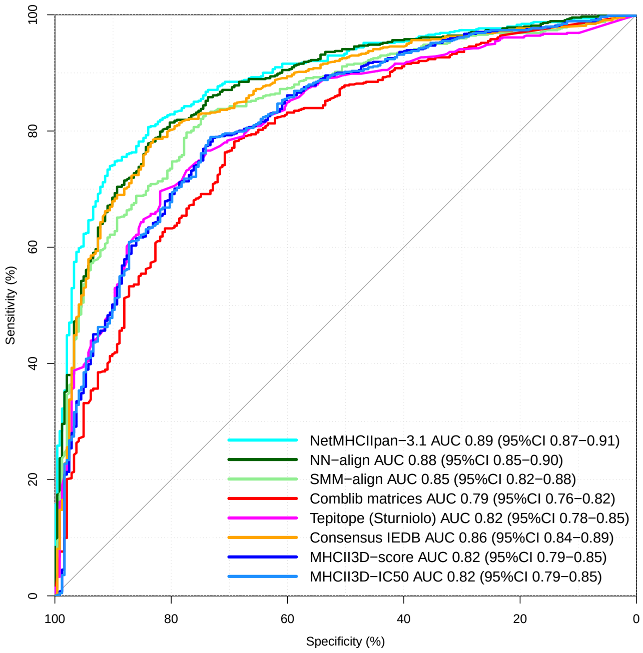

2016-12-31 - 1028057

DRB1*01:01 - #peptides/#binder: 29/22

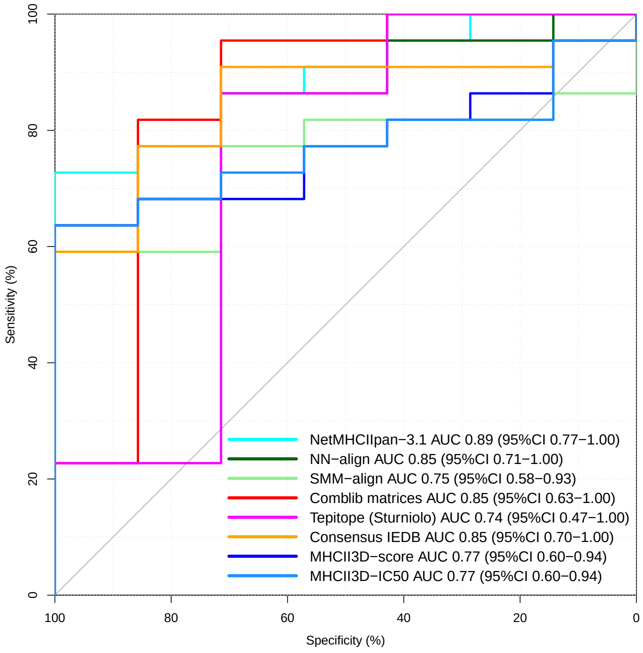

2016-12-31 - 1028057

DRB1*04:01 - #peptides/#binder: 29/25

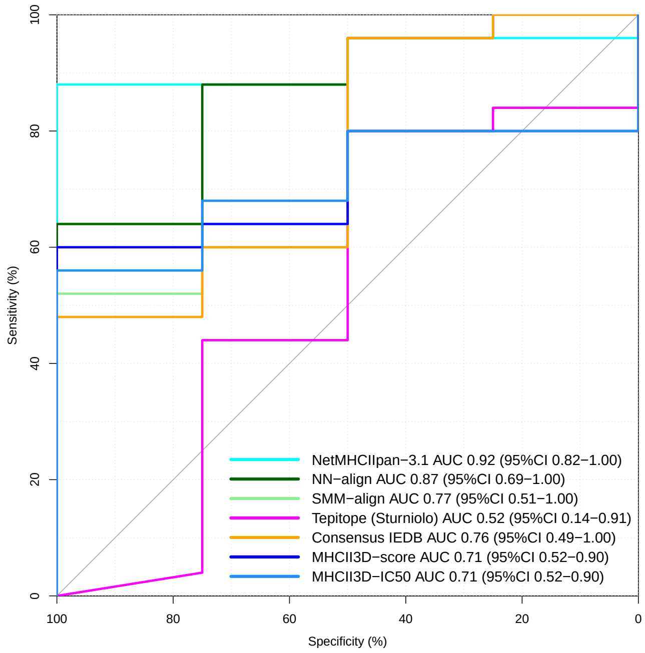

2016-12-31 - 1028057

DRB1*07:01 - #peptides/#binder: 29/27

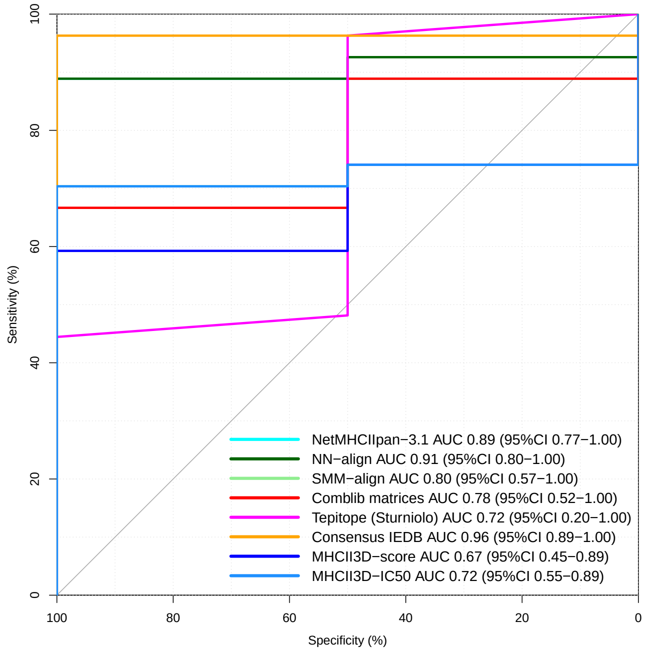

2016-12-31 - 1028057

DRB1*15:01 - #peptides/#binder: 29/26

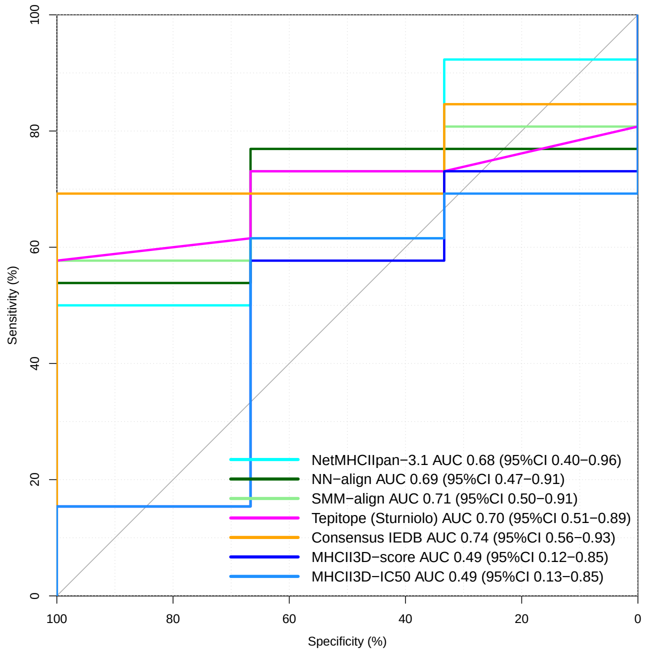

2016-12-31 - 1027578

DRB1*03:01 - #peptides/#binder: 14/10

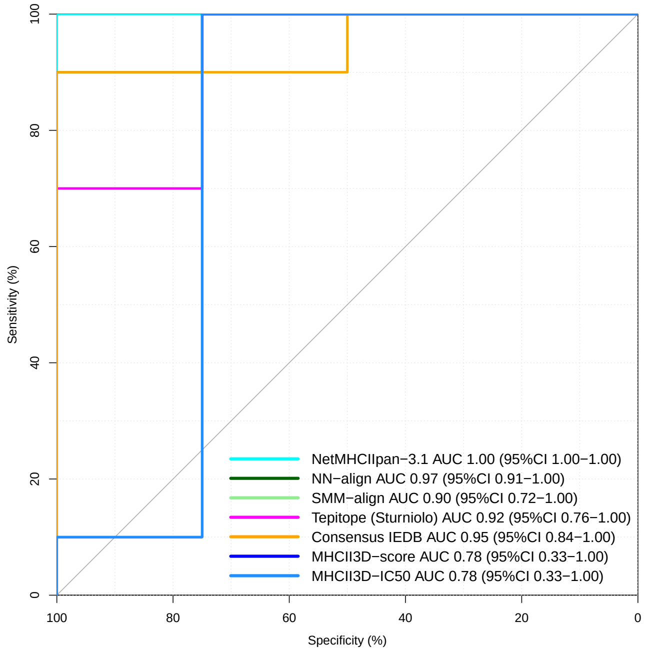

2016-12-31 - 1027578
DRB1*07:01 - #peptides/#binder: 19/12

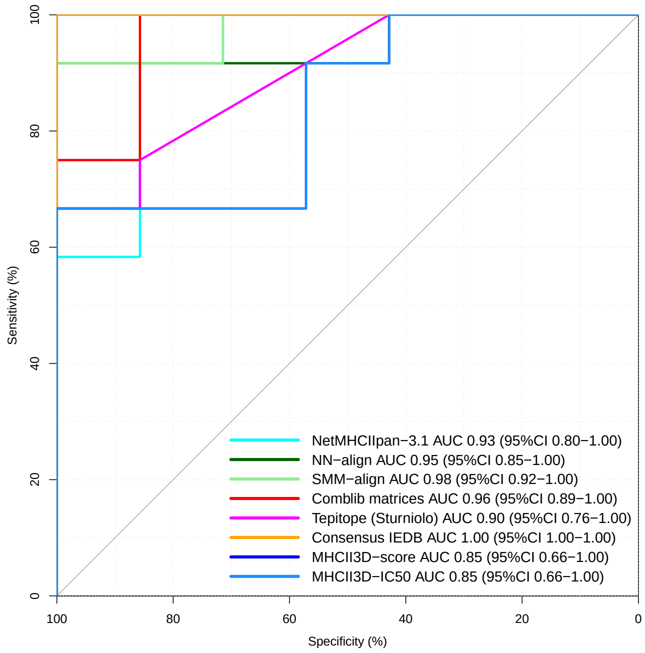

2016-12-31 - 1027578
DRB3*01:01 - #peptides/#binder: 20/7

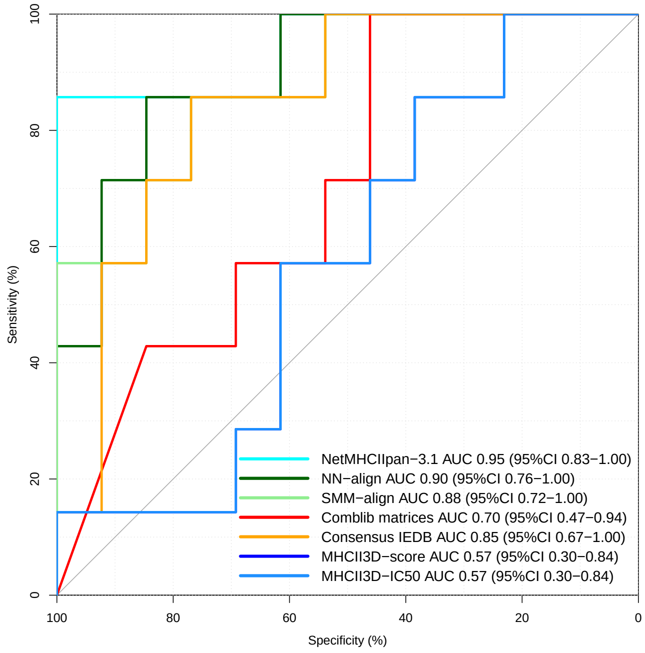

2016-12-31 - 1027578

DRB4*01:01 - #peptides/#binder: 14/4

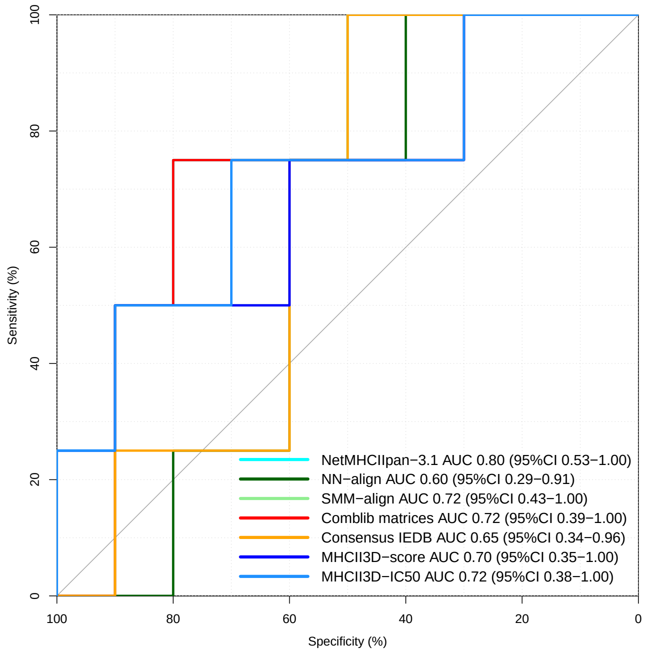

2017-11-24 - 1032311

DRB1*01:01 - #peptides/#binder: 16/14

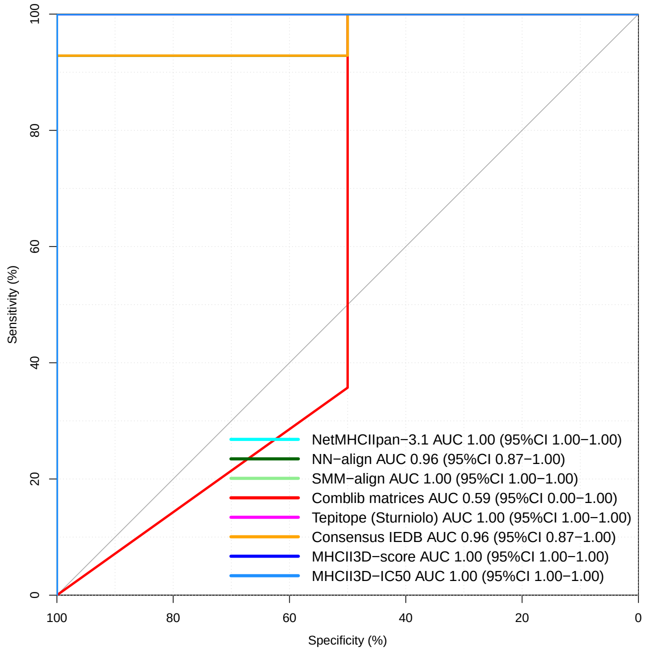

2018-11-23 - 1029531

DRB1*01:01 - #peptides/#binder: 11/4

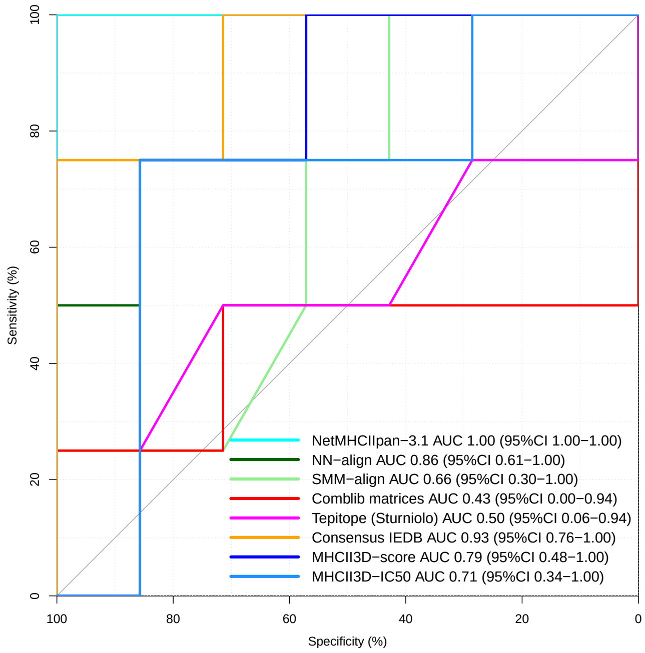

2019-03-22 - 1034502

DRB1*03:01 - #peptides/#binder: 21/3

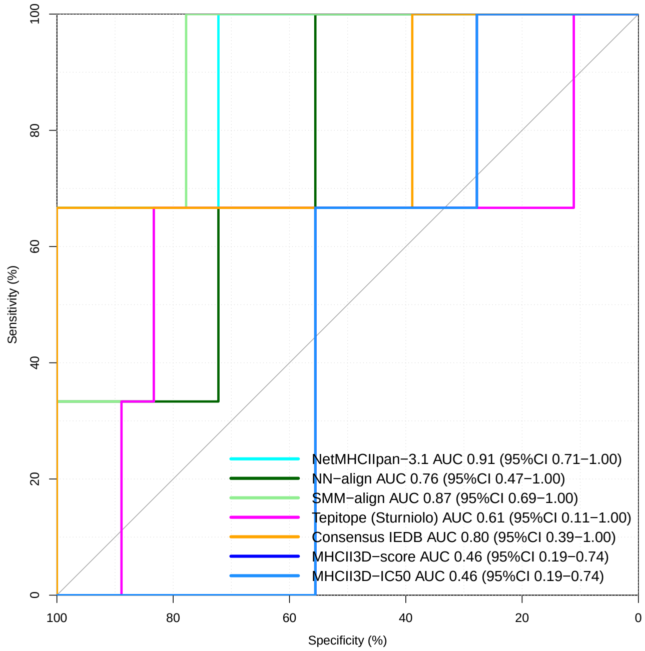

2019-03-22 - 1034502

DRB1*08:02 - #peptides/#binder: 21/5

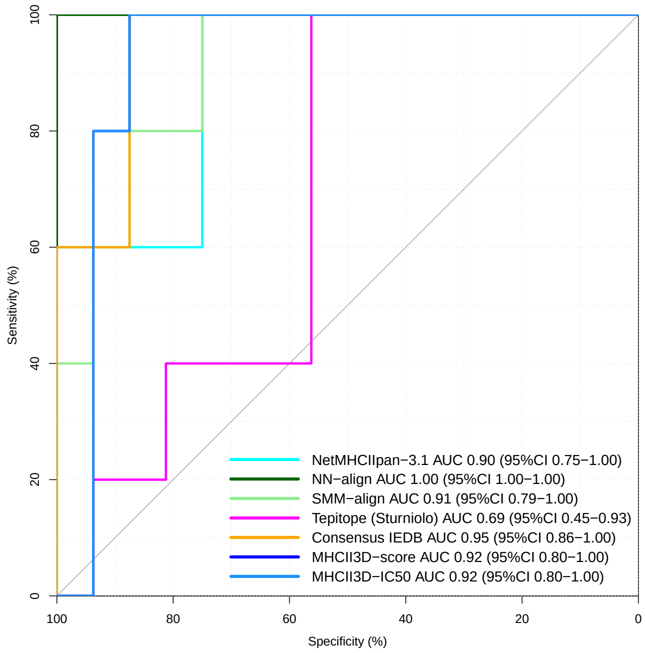

2019-03-22 - 1034502

DRB1*11:01 - #peptides/#binder: 21/5

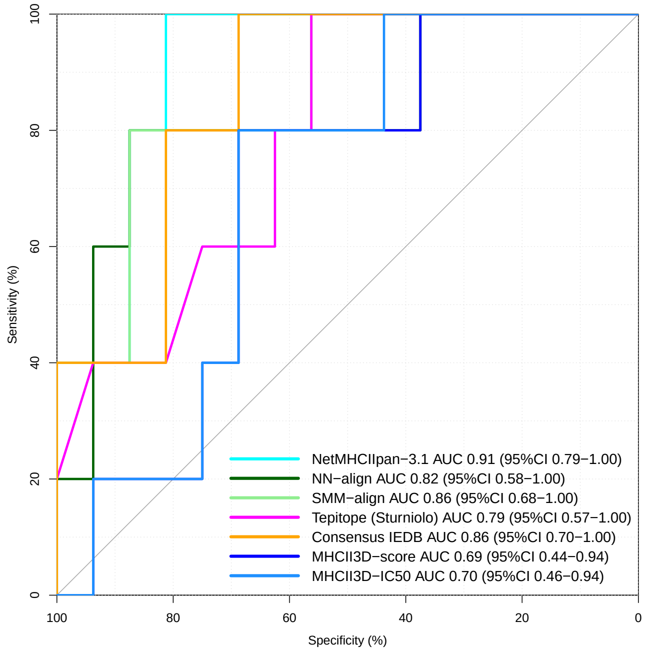

2019-03-22 - 1034502

DRB1*15:01 - #peptides/#binder: 21/4

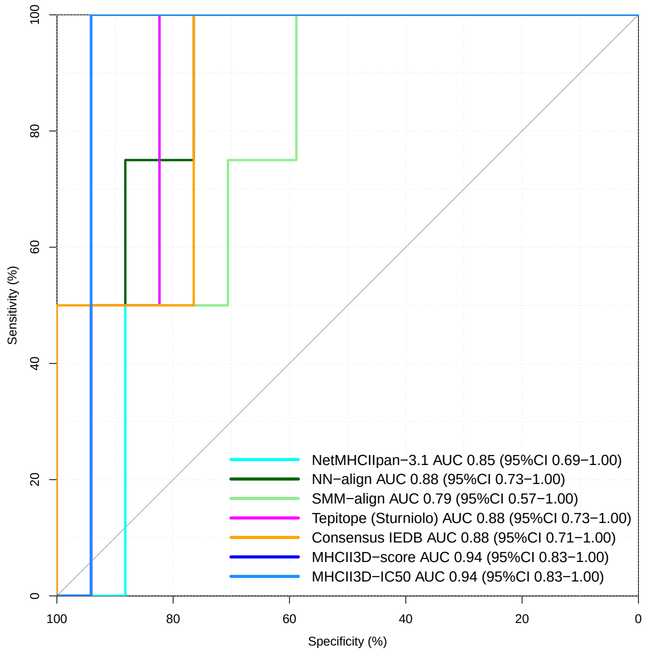

Supplement: Supplementary file 1 [file ijms-22-00012-s001.zip › S1_table1_ROC.pdf]
