# Supplementary material for: MHCII3D—Robust Structure Based Prediction of MHC II Binding Peptides
Source: Int J Mol Sci. 2020 Dec 22;22(1):12. doi: 10.3390/ijms22010012 (PMC7792572; doi:10.3390/ijms22010012)
Supplement: Supplementary file 1 [file ijms-22-00012-s001.zip › S4_contradictingEntries.pdf]

S4 - Contradicting Entries

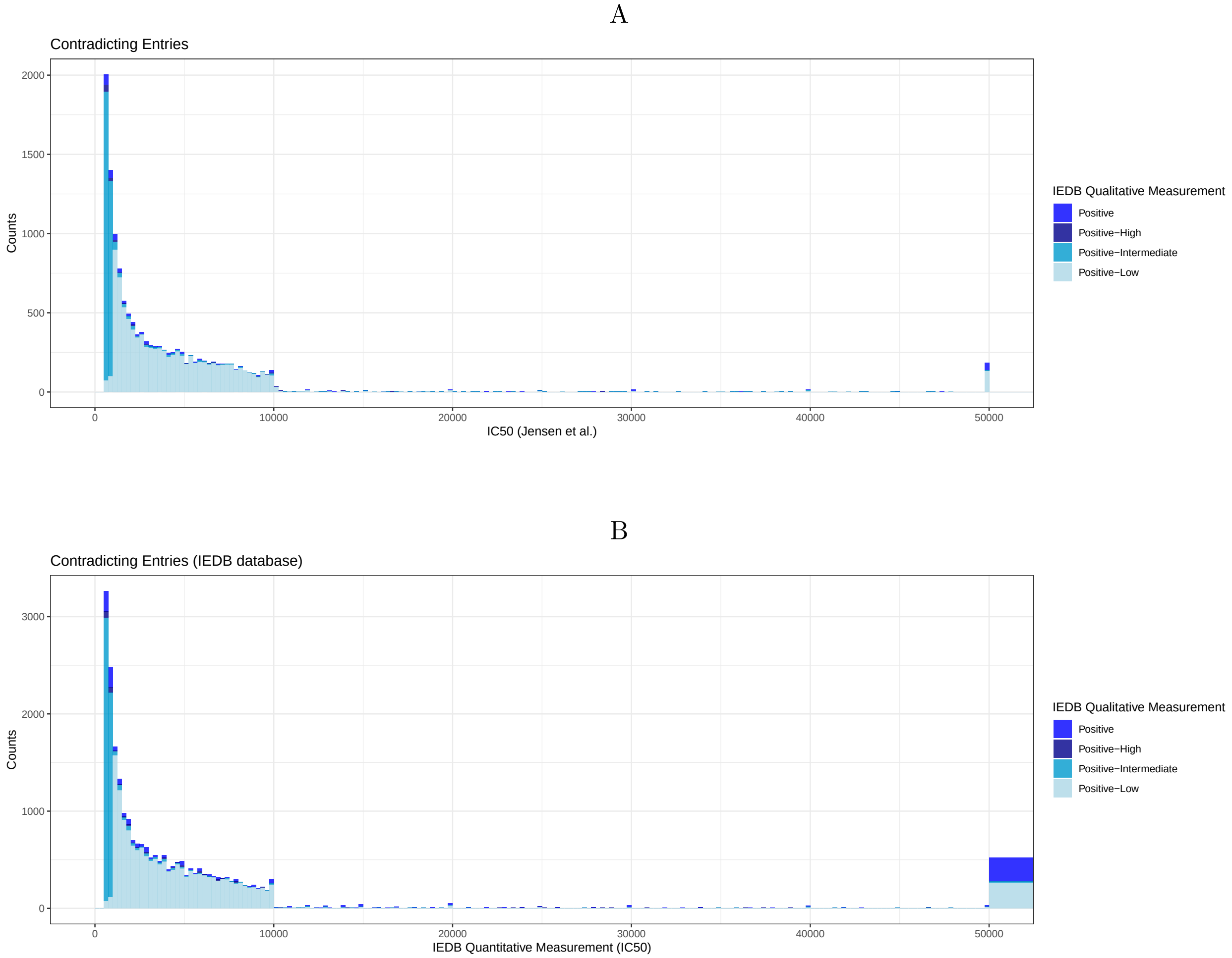

Figure S4: Contradicting IEDB entries. In many cases, the qualitative assessment of IEDB entries contradicts the presented quantitative measurement (IC_{50} cutoff 500nM). The histograms show the IC_{50} distribution of these entries, for (A) a set of 35529 entries which can be mapped between the IEDB database and a data set provided by Jensen *et al.* [1], and (B) for all IEDB entries providing an IC_{50} value. Entries with an inequality flag were excluded from this analysis.

References

[1] Jensen KK, Andreatta M, Marcatili P, Buus S, Greenbaum JA, Yan Z, Sette A, Peters B, Nielsen M. Improved methods for predicting peptide binding affinity to MHC class II molecules. *Immunology* 2018
