# Supplementary material for: MHCII3D—Robust Structure Based Prediction of MHC II Binding Peptides
Source: Int J Mol Sci. 2020 Dec 22;22(1):12. doi: 10.3390/ijms22010012 (PMC7792572; doi:10.3390/ijms22010012)
Supplement: Supplementary file 1 [file ijms-22-00012-s001.zip › S8_number_alternative_peptides.pdf]

S8 - Effect of the number alternative peptide conformations

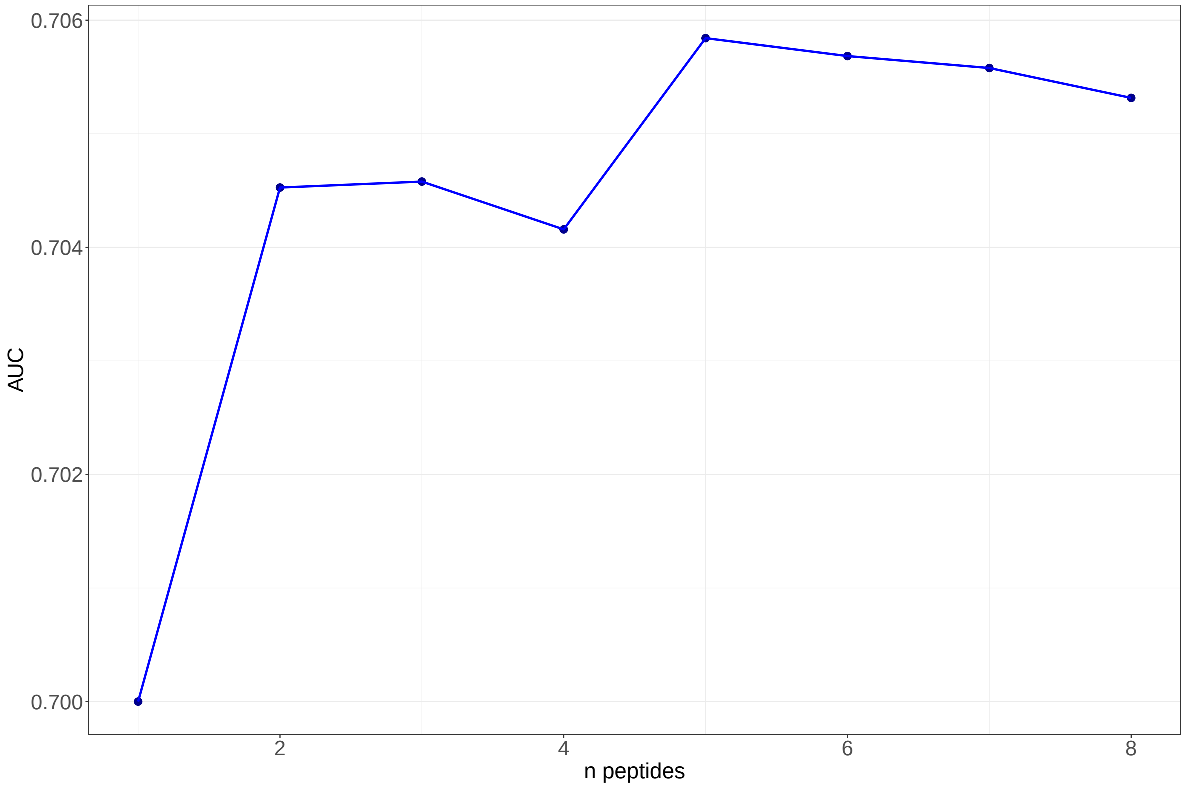

Figure S8: The number of alternative peptide conformations: Effect of various numbers of alternative peptide conformations on classification performance. During the prediction, a number of alternative peptide conformations are used to obtain small conformational varieties. These small variations positively affect the prediction performance in comparison to predictions on a single model. We optimized the number of alternative peptide conformations on entries of the IEDB weekly benchmarks sets (`http://tools.iedb.org/auto_bench/mhcii/weekly/`) for which no prediction values of other methods are provided and therefore were not used in other analyses. The plot shows the average classification performance per allele for various numbers of alternative peptide conformations.
